# Guanxinping Tablets Inhibit ET-1-Induced Proliferation and Migration of MOVAS by Suppressing Activated PI3K/Akt/NF-*κ*B Signaling Cascade

**DOI:** 10.1155/2022/9485463

**Published:** 2022-05-31

**Authors:** Ying-Ying Wu, Yi-Lin Huang, Bin Dai, Jian-Wei Liu, Xu Han

**Affiliations:** ^1^Suzhou TCM Hospital Affiliated to Nanjing University of Chinese Medicine, Suzhou 215000, China; ^2^Jiangsu Province Hospital of Chinese Medicine, Affiliated Hospital of Nanjing University of Chinese Medicine, Nanjing 210029, China

## Abstract

**Background/Aim:**

Abnormal proliferation and migration of vascular smooth muscle cells is one of the main causes of *atherosclerosis* (AS). Therefore, the suppression of abnormal proliferation and migration of smooth muscle cells are the important means for the prevention and inhibition of AS. The clinical effects of Guanxinping (GXP) tablets and preliminary clinical research on the topic have proved that GXP can effectively treat coronary heart disease, but its underlying mechanism remains unclear. This study aimed to confirm the inhibitory effect of GXP on the abnormal proliferation of mouse aortic vascular smooth muscle (MOVAS) cells and to explore the underlying mechanism.

**Methods:**

MOVAS cells were divided into two major groups: physiological and pathological groups. In the physiological group, MOVAS cells were directly stimulated with GXP, whereas in the pathological group, the cells were stimulated by endothelin-1 (ET-1) before intervention by GXP. At the same time, atorvastatin calcium, which effectively inhibits the abnormal proliferation of MOVAS cells, was used in the negative control group. CCK8 assay, scratch test, ELISA, Western blotting, and immunofluorescence staining were performed to observe the proliferation and migration of MOVAS cells and the expression levels of related factors after drug intervention in each group.

**Results:**

In the physiological group, GXP had no significant effect on the proliferation and migration of MOVAS cells and the related factors. In the pathological group, a high dose of GXP reduced the abnormal proliferation and migration of MOVAS cells. Further, it reduced the expression levels of PI3K; inhibited the phosphorylation of Akt (protein kinase B); upregulated I*κ*B-*α* levels; prevented nuclear factor kappa B (NF-*κ*B) from entering the nucleus; downregulated the expression of interleukin 6 (IL6), IL-1*β*, and iNOS; and upregulated the ratio of apoptosis-related factor Bax/Bcl-2. There was no significant difference between the high-dose GXP group and the atorvastatin calcium group (negative control group).

**Conclusion:**

Our findings revealed that GXP was able to inhibit the proliferation and migration of MOVAS cells by regulating the PI3K/Akt/NF-*κ*B pathway.

## 1. Introduction

The proliferation and migration of vascular smooth muscle cells (VSMCs) is one of the main causes of atherosclerosis (AS). Under physiological conditions, VSMCs regulate vascular tension and blood pressure. VSMCs proliferate and migrate excessively and secrete inflammatory factors under conditions of vascular injury, inflammation, and oxidative stress [1]. Therefore, we only need to inhibit the proliferation and migration of smooth muscle cells in the pathological state but not in the physiological state.

ET-1 is a peptide synthesized by endothelial cells, which can accelerate the AS process. In the physiological state, ET-1 releases nitric oxide (NO) and prostacyclin by binding to endothelin type A (ETA) and endothelin B (ETB) receptors, thus regulating the relaxation, proliferation, and migration of VSMCs. In a pathological state, ETA and ETB receptors are highly expressed in vascular smooth muscles, stimulating smooth muscle cells to contract, proliferate, and migrate [2, 3]. Atorvastatin calcium is one of the 3-hydroxy-3-methylglutaryl coenzyme A reductase inhibitors, which plays an antiatherosclerotic role by reducing plasma lipids and inhibiting intimal smooth muscle cell accumulation [4]. Further, atorvastatin calcium can inhibit the proliferation and migration of VSMCs by inhibiting PI3K/Akt signaling pathway but has no obvious effect on VSMCs in a physiological state [5].

Akt, a member of serine threonine kinase, has three different subtypes: Akt1, Akt2, and Akt3, which play an important role in cell proliferation and survival [6]. PI3K is the upstream signaling factor of Akt, which promotes Akt phosphorylation by activating t308 and s473. PI3K/Akt can affect the process of AS by regulating the proliferation and migration of smooth muscle cells [7]. The activation of PI3K/Akt signaling can affect macrophage apoptosis by promoting antiapoptotic factors Bcl-xl and Bcl-2 and inhibiting proapoptotic factor Bcl-2-associated *X* (Bax); thus, it is a key determinant of the number of atherosclerotic plaque cells [8]. Furthermore, NF-*κ*B, a key nuclear factor, which usually combines with inhibitor of *κ*B (I*κ*B) to maintain an inactive state, can mediate cell proliferation and apoptosis [9]. NF-*κ*B is implicated in multiple pathological processes of atherogenesis, such as inflammation, oxidative stress, and VSMC proliferation [10]. Accordingly, the expression of NF-*κ*B increases when PI3K/Akt signaling pathway is activated [11].

GXP is the experience prescription by Qi-yi Li, a famous Chinese Medicine professor. Professor Li summed up the theory of *Gualou xiebai baijiu decoction* in the synopsis of prescriptions of the golden chamber and the Danggui buxue decoction in the differentiation of endogenous and exogenous diseases. Gualou xiebai baijiu decoction and Danggui buxue decoction have been widely used to treat coronary heart disease.

**Figure 1:**
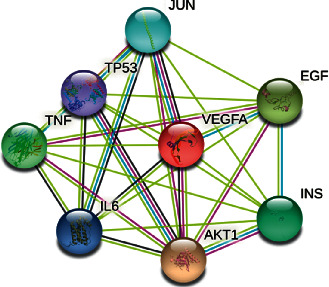
PPI network of GXP in the treatment of *atherosclerosis.*

Subsequently, Li added and removed two prescriptions to get GXP. GXP is a composition of five herbs, including Polygonatum sibiricum, Angelica sinensis, Panax notoginseng (Burkill) F.H.Chen, Trichosanthes kirilowii Maxim. Peel, and Nardostachys chinensis, and has been used clinically for decades; it has also been approved as a hospital preparation since 2001. The quality of GXP can be controlled [12, 13]. Through analyses via LC-MS/MS and LCMS-Q-TOF methods, it can be concluded that in the charge quality comparison plot (M/Z: 200∼750), there are many components and concentrations of ethyl acetate extracted species, especially Nardostachys chinensis and Trichosanthes kirilowii Maxim. Peel, in the large range of charge mass ratio (M/Z: 750∼1200); the n-butanol extract has more components and higher concentrations of particularly Angelica sinensis and Panax notoginseng (Burkill) F.H.Chen [14]. The effective components of GXP are mainly Notoginsenoside R1, Ginsenoside Rg1, and Ginsenoside Rb1 [12]. Previous network pharmacological studies have shown that GXP is closely associated with Akt and IL-6 (Table 1, Figure 1). Combined with previous studies, it can be seen that GXP can reduce the expression of NF-*κ*B. Accordingly, we next speculated whether the antiatherosclerotic effect of GXP is associated with the PI3K/Akt/NF-*κ*B pathway. Thus, we studied the relationship between the effect of GXP on the proliferation and migration of VSMCs and PI3K/Akt/NF-*κ*B pathway [15].

## 2. Materials and Methods

### 2.1. Animal and Preparation of Medicated Serum

All experimental protocols in this study were approved by the Ethics Committee of Jiangsu Province Hospital of Chinese Medicine. All procedures were performed in accordance with ethical standards and laboratory care by the National Institute of Health, China. 6-week-old male Sprague-Dawley rats, weighing 200–220 g, were purchased from Hangzhou Medical College, Zhejiang, CHN (SCXK2019-0002). The dosage for each mouse was according to the human and rat dosage conversion formula: rat dosage (mg/kg) = human (70 kg) dosage × 0.018/0.2 kg. GXP group was given GXP tablets (2.43 g/kg·bid, Jiangsu Province Hospital of Chinese Medicine, CHN), atorvastatin calcium group was given atorvastatin calcium (0.018 g/kg·bid, Lepu, CHN), control group was given equal volume of distilled water [16, 17]. Fasting for 12 h after 3 days of intragastric administration, and 1 h after intragastric administration on the 4th day, mice were anesthetized by inhalation of ether. Blood was taken from the abdominal aorta to obtain medicated serum. During the experiment, every effort was made to minimize the pain of animals [18].

### 2.2. Cell Culture

Mouse aortic vascular smooth muscle cells (MOVAS, ACTT number:CRL-2797) and FBS were purchased from Cellcook Biotech Co., China. MOVAS were cultured in DMEM (Gibco, USA) medium containing 10% FBS, 0.2 mg/ml G418 (BioFroxx, GER), penicillin, and streptomycin (Gibco, USA, 1 ： 100) at 37°C and 5% CO_2_. The cells were divided into physiological group and pathological group. The physiological group included the control group (blank group), low- and high-dose GXP groups, and atorvastatin calcium group. The pathological group included endothelin-induced ET-1 group (model group), ET-1 +  low- and high-dose GXP groups, and ET-1 + atorvastatin calcium group.

### 2.3. Safety and Toxicity Assay

For the CCK-8 assay, cells were seeded in 96-well culture plates (4000 cells/well). All these plates were shaked back and forth with a figure “eight” to distribute the cells evenly and then incubated for 12 h in a 37°C incubator to make them adherent. The media of each group were replaced with the medium containing 2% FBS （treated with serum starvation） for 12 h. Then, 96-well culture plates were divided into four groups. Each group was added separately with 1%, 10%, and 20% medicated serum of GXP or 10% medicated serum of atorvastatin calcium. After that, the original mediums were replaced by mediums containing 10% CCK-8. An automatic microplate reader (BIOTEK, USA) was used to determine the absorbance at 490 nm when 0 h, 12 h, 24 h, 48h, and 72 h incubation in the dark.

### 2.4. Cell Viability Assay

For the CCK-8 assay, cells were seeded in 96-well culture plates (4000 cells/well). All these plates were shaked back and forth with a figure “eight” to distribute the cells evenly and then incubated for 12 h in a 37°C incubator to make them adherent. The media of each group were replaced with the medium containing 2% FBS （treated with serum starvation) for 12 h. Then, each well of the pathological group was intervented with ET-1 (10^-4 mmol/L, Aladdin CHN) [19] for 24 h. Then, the blank group and the model group were added with 10% blank rat serum. The low- and high-dose GXP groups were added with 1% and 10% medicated serum of GXP. The ET-1 + low- and high-dose GXP groups were added with 1% and 10% medicated serum of GXP as well. Both atorvastatin calcium group and ET-1 + atorvastatin calcium group were added with 10% medicated serum of atorvastatin calcium. The corresponding serum stimulated each group for 48 hours. After 48 h, the original mediums were replaced by mediums containing 10% CCK-8. An automatic microplate reader (BIOTEK, USA) was used to determine the absorbance at 490 nm after 3 h incubation in the dark [20].

### 2.5. Wound Healing Assay

A straight line was drawn on the bottom of the culture dish. MOVAS were subcultured in 8 culture dishes. MOVAS were treated with serum starvation for 24 h after reaching about 80% of the culture dishes. After that, the media of each group were replaced with the medium containing 2% FBS （treated with serum starvation) for 12 h. In the pathological group, MOVAS were treated with ET-1 for 24 hours. The 200 *μ*l sterile pipette tip was used to make a scratch along the ruler, which was perpendicular to the straight line of the bottom. Then, the blank group and the model group were added with 10% blank rat serum. The low- and high-dose GXP groups were added with 1% and 10% medicated serum of GXP. The ET-1 + low- and high-dose GXP groups were added with 1% and 10% medicated serum of GXP as well. Both atorvastatin calcium group and ET-1 + atorvastatin calcium group were added with 10% medicated serum of atorvastatin calcium. The corresponding serum stimulated each group for 48 h. The scratch conditions were photographed at 0 h, 24 h, and 48 h, and the scratch area was calculated by Image J 1.53 software (NIH, USA) [21].

### 2.6. ELISA Assay

Same as the previous experiments, MOVAS were treated with starvation for 12 h. ET-1 inducted MOVAS of the pathological group for 24 h. And then, the corresponding drugs stimulated each group for 48 h. Then, the supernatant of the cell culture medium was collected. The expression of inflammatory factors (IL-6, IL-1*β*, and TNF-*α*) levels in the cell culture medium was assayed by ELISA kit (Thermo Fisher Scientific, USA) according to the instructions [22].

### 2.7. Western Blot Analysis

Same as the previous experiments, MOVAS were treated with starvation for 12 h. ET-1 inducted MOVAS of the pathological group for 24 h. And then, the corresponding drugs stimulated each group for 48 h. Protein was extracted from lysed cells. When we detected NF-*κ*B, we extracted the nuclear protein from MOVAS. While assaying other factors, the total protein was extracted. The SDS-PAGE gel was prepared with a gel preparation kit (Servicebio, CHN). The samples of each group were separated by SDS gel electrophoresis and then transferred to nitrocellulose membranes, added the primary antibodies overnight after blocking and incubation, and incubated with secondary antibody for 1 h the next day. Protein signals were visualized by ChemiDoc System (BioRad). ImageJ 1.53 software was used to calculate the gray value. The main antibodies used in this study are anti-PI3K (Proteintech, CHN, 1 : 3000), anti-Akt (Proteintech, 1 : 1000), anti-phospho-Akt (Proteintech, 1 : 2000), anti-I*κ*B-*α* (Cell Signaling Technology, USA, 1 : 1000), anti-NF-*κ*B (Proteintech, 1 : 2000), anti-Bax (CST, 1 : 1000), anti-Bcl-2 (Proteintech, 1 : 1000), anti-GAPDH (Proteintech, 1 : 5000), anti-H3 (Proteintech, 1 : 1000), anti-iNOS (CST, 1 : 1000), anti-TNF-*α* (CST, 1 : 1000), anti-Rabbit IgG (Servicebio, 1 : 3000), and anti-Mouse IgG (Servicebio, 1 : 3000) [23, 24].

### 2.8. Immunofluorescence Staining

MOVAS were inoculated in a 12-well plate. After 12 h of culture, cells were treated in the same way as the previous experiment. Both the physiological group and the pathological group were treated with starvation for 12 h. ET-1 inducted MOVAS of the pathological group for 24 h. And then, the corresponding drugs stimulated each group for 48 h. After fixing, rupturing, and blocking, we added the primary antibody (anti-NF-*κ*B, Proteintech, 1 : 500) in a plate overnight at 4°C, then incubated with fluorescent secondary antibody (anti-Rabbit IgG, Servicebio, 1 : 400) for 1 h at room temperature and dark. Incubated with DAPI (Servicebio) at room temperature for 10 min, mount the slide with antifluorescence quenching solution. The fluorescence photos were photographed under fluorescent microscopy (NIKON, JPN), and then, ImageJ 1.53 software was used to count the fluorescence brightness [25].

### 2.9. Statistical Analysis

Each of the above experiments was carried out three times or more independently repeated experiments. Data are reported as the mean ± standard deviation (SD). The data of different magnitude was normalized and subjected to one-way analysis of variance (ANOVA) and used SPSS 25.0 statistical software (IBM, USA). *P*-value <0.05 was considered significant.

## 3. Results

### 3.1. Safety and Toxicity of GXP

**Figure 2:**
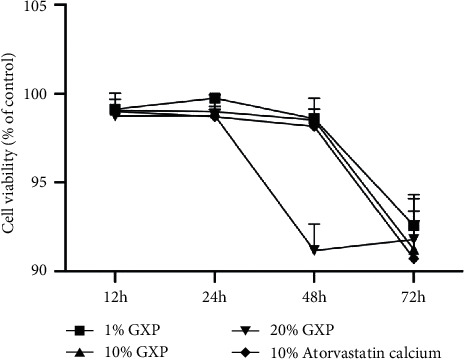
The time concentration gradient of medicated serum intervention MOVAS determined by the CCK8 method. GXP represents medicated serum of GXP, and atorvastatin calcium represents medicated serum of atorvastatin calcium.

The safety and toxicity of GXP were measured by CCK8. Cell viability at different concentrations and intervention times showed that 1% and 10% medicated serum of GXP and 10% medicated serum of atorvastatin calcium showed no obvious toxicity within 48 hours (Figure 2).

### 3.2. Effect of GXP on the Survival Rate of MOVAS

**Figure 3:**
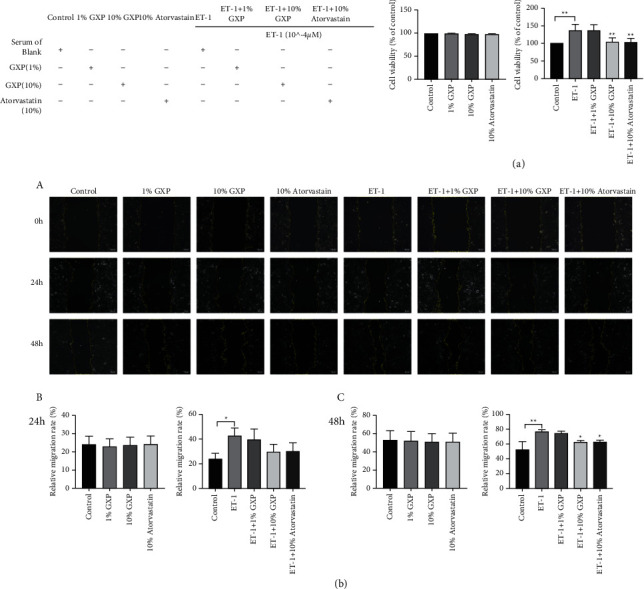
Effects of GXP on the proliferation and migration of MOVAS. (a) CCK-8 assay showed that GXP inhibited the excessive proliferation of MOVAS which was raised by ET-1 while GXP had no effect on the physiological group; (b) wound-healing assay showed that GXP inhibited the excessive migration of MOVAS which was raised by ET-1. But GXP had no effect on the physiological group 200x. (*A*) Pictures taken in wound-healing assay. In the physiological group, other groups were compared with the control group, and in the pathological group, other groups were compared with the model group (ET-1 group). (*B*) 24 h relative migration rate of MOVAS; (*C*) 48 h relative migration rate of MOVAS, ^*∗*^*P* < 0.05, ^*∗∗*^*P* < 0.01 compared with the model group (ET-1 group).

In this experiment, CCK8 assay was used to measure the cell survival rate of each group (Figure 3(a)). In the physiological group, there was no significant difference in cell survival rate between each group. It indicated that GXP had no significant effect on cell survival rate under the physiological state of MOVAS. The survival rate of the cells was significantly increased after stimulation with ET-1 (cell viability, 137.6 ± 17.14%, *n* = 3 (*P* < 0.01 as control)). It showed that ET-1 led to abnormal proliferation of MOVAS. After the treatment of the high dose of GXP, the survival rate of cells decreased significantly (cell viability, 105.1 ± 10.91%, *n* = 3 (*P* < 0.01 as model)). There was no significant difference between the high-dose GXP group and the atorvastatin calcium group. The results showed that high-dose GXP could inhibit the abnormal proliferation of MOVAS, and its effect was no less than atorvastatin calcium.

### 3.3. Effect of GXP on the Mobility of MOVAS

The mobility of MOVAS was evaluated by wound healing assay (Figure 3(b)). According to the formula, 24 h mobility = (0 h scratch area − 24 h scratch area)/0 h scratch area, and the mobility of each group was calculated. At the 24th hour, the migration rate of the model group was higher than the control group (relative migration rate, 42.78 ± 6.201%, *n* = 3 (*P* < 0.05)). But, there was no significant difference among the other groups (Figure 2(a)). At the 48th hour, there was still no difference among each group in the physiological group. In the pathological group, the mobility of the high-dose GXP group (relative migration rate, 62.94 ± 1.799%, *n* = 3 (*P* < 0.05 as model)) and atorvastatin calcium group (relative migration rate, 63.39 ± 1.809%, *n* = 3 (*P* < 0.05 as model)) was lower than that of the model group. There was no significant difference between the high-dose GXP group and the atorvastatin calcium group (Figure 2(b)). These results proved that high-dose GXP inhibited the excessive migration of MOVAS, and its effect was no less than that of atorvastatin calcium. At the same time, it was verified that the proliferation and migration of smooth muscle cells could be induced after being stimulated by ET-1 for 24 hours [19] while drugs needed 48 hours to stimulate the cells before they had an obvious effect. That provided the basis for the later experiment.

### 3.4. Effect of GXP on the Expression of Inflammatory Factors in MOVAS

**Figure 4:**
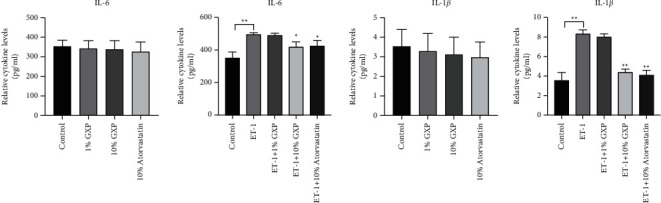
Effect of GXP on the expression of inflammatory factors in MOVAS. In the physiological group, other groups were compared with the control group, and in the pathological group, other groups were compared with the model group (ET-1 group), ^*∗*^*P* < 0.05, ^*∗∗*^*P* < 0.01 compared with the model group (ET-1 group).

ELISA assay was used to test the expression of IL-6, IL-1*β*, and TNF-*α*. The results showed that the levels of IL-6 and IL-1*β* in the physiological group were similar. In the pathological group, IL-6 (relative cytokine levels, 498.8 ± 8.128, *n* = 3 (*P* < 0.01)) and IL-1*β* (relative cytokine levels, 8.353 ± 0.3811, *n* = 3 (*P* < 0.01)) in the model group were much higher than those in the control group. High-dose GXP group and atorvastatin calcium group decreased the expression of IL-6 (relative cytokine levels, 422.8 ± 29.32, 428.2 ± 34.74, *n* = 3 (*P* < 0.05 as model)) and IL-1*β* (relative cytokine levels, 4.443 ± 0.2870, 4.130 ± 0.4727, *n* = 3 (*P* < 0.05 as model)) (Figure 4). TNF-*α* was measured more than three times, but no results have been detected yet. In this study, for the moment, TNF-*α* was not considered its effect on the proliferation and migration of MOVAS.

### 3.5. Effect of GXP on PI3K/Akt/NF-кB Pathway on the Expression of Related Proteins

**Figure 5:**
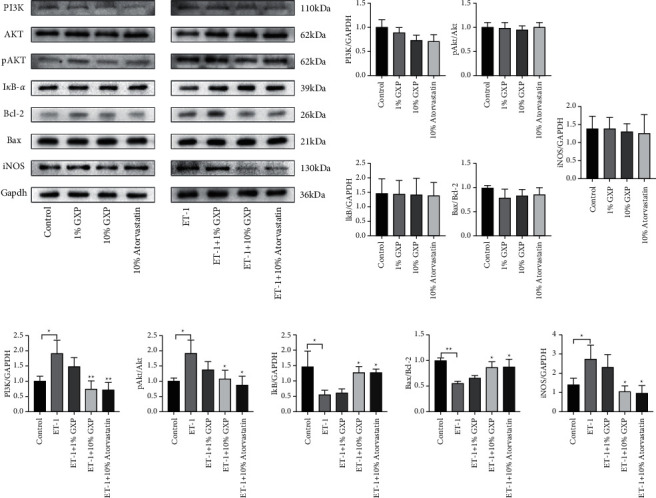
Effect of GXP on PI3K/Akt pathway on the expression of related proteins. GXP inhibited the activation of ET-1 to PI3K/AKT signaling pathway. The protein expression levels of PI3K, Akt, pAkt, I*κ*B-*α*, Bax/Bcl-2, and iNOS were determined by Western blotting. The graphs represent the relative activity of these proteins for three independent experiments. (a) The band and molecular weight of each protein in Western blotting; (b) the relative expression of each protein in the physiological group; (c) the relative expression of each protein in the pathological group. In the physiological group, other groups were compared with the control group, and in the pathological group, other groups were compared with the model group (ET-1 group),^*∗*^*P* < 0.05, ^*∗∗*^*P* < 0.01 compared with the model group (ET-1 group).

We detected the level of PI3K/Akt/NF-*κ*B pathway-related proteins by Western blot analysis (Figure 5). Besides, we also observed the nuclear translocation of NF-*κ*B by immunofluorescence staining. We found that there was no significant difference in protein expression in the physiological group (Figure 5(b)). ET-1 activated PI3K/Akt/NF-*κ*B pathway and gave rise to the changes in related proteins. In the pathological group, GXP reduced the expression of PI3K (0.7602 ± 0.2543, *n* = 3 (*P* < 0.01 as model)), inhibited the phosphorylation of Akt (1.095 ± 0.2712, *n* = 3 (*P* < 0.05 as model)), increased the level of I*κ*B-*α* expression (1.287 ± 0.1881, *n* = 3 (*P* < 0.05 as model)), upregulated Bax/Bcl-2 ratio (0.8657 ± 0.1050, *n* = 3 (*P* < 0.05 as model)), and decreased the expression of iNOS (1.064 ± 0.2859, *n* = 3 (*P* < 0.05 as model)) (Figure 5(c)).

### 3.6. Effect of GXP on the Entry of NF-*κ* to the Nucleus

**Figure 6:**
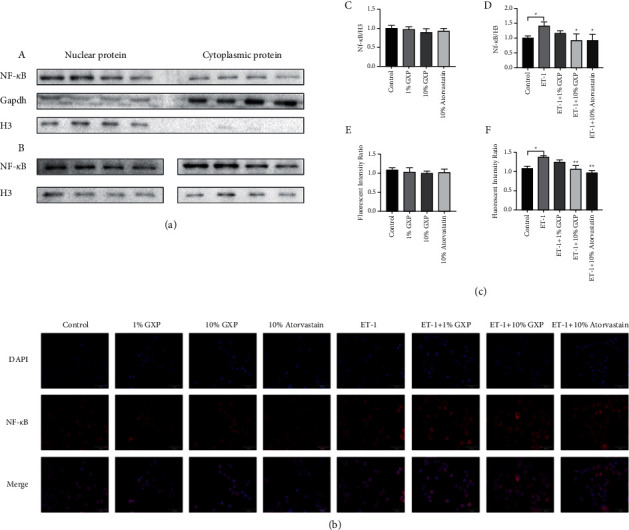
Effect of GXP on the expression of NF-*κ*B. GXP inhibited the activation of ET-1 on NF-*κ*B signaling pathway. The graphs represent the level of NF-*κ*B for three independent experiments. (a) The protein expression levels of NF-*κ*B. (*A*) Comparison of NF-*κ*B, GAPDH, and H3 protein expression in nucleus and cytoplasm. (*B*) The band of NF-*κ*B protein in Western blotting; (b) immunofluorescence staining was performed to determine the expression and distribution of NF-*κ*B, 400x. The expression of NF-*κ*B was red, and nuclei were stained blue with DAPI. The micrographs are representative of three independent experiments; (c) (*C*, *D*) the relative expression of NF-*κ*B protein in physiological and pathological groups. (*E*, *F*) The ratio of NF-*κ*B fluorescence intensity in physiological and pathological groups. In the physiological group, other groups were compared with the control group, and in the pathological group, other groups were compared with the model group (ET-1 group), ^*∗*^*P* < 0.05, ^*∗∗*^*P* < 0.01 compared with the model group (ET-1 group).

After stimulated by ET-1, the expression of NF-*κ*B (1.416 ± 0.1223, *n* = 3 (*P* < 0.05)) and fluorescence intensity (fluorescence intensity ratio, 1.378 ± 0.0369 *n* = 3 (*P* < 0.01)) in the nucleus increased. It showed that the level of NF-*κ*B in the nucleus increased. After intervented by GXP (0.9309 ± 0.2151, *n* = 3 (*P* < 0.05)) (fluorescence intensity ratio, 1.070 ± 0.0893 *n* = 3 (*P* < 0.01)) or atorvastatin calcium, the fluorescence intensity in nucleus decreased. This proved that GXP inhibited the activation of NF-*κ*B by ET-1 (Figure 6).

## 4. Discussion

Many vascular diseases such as coronary heart disease, stroke, and intermittent claudication are all results of AS. Despite major advances in prevention measures, many individuals die of acute complications of AS [26]. Therefore, it is very significant to make further efforts to study the preventive measures of AS. VSMCs are an important part of blood vessels and are closely associated with AS. Endogenous levels of oxidative DNA damage in VSMCs promote atherosclerotic plaque formation [27]. VSMCs accelerate the formation of atherosclerotic plaques in the state of premature aging, which is induced by replicative failure and stress [28]. In addition, the dysregulation of the autophagy level of smooth muscle cells deteriorates AS [29]. Endogenous DNA oxidative damage, premature aging, and autophagy all affect the proliferation and migration of smooth muscle cells and aggravate AS. Nevertheless, VSMCs regulate vascular tension and blood pressure without obvious proliferative changes under physiological conditions [1].

In the past decades, more than 1,000 clinical studies on GXP have been conducted. They have proven the efficacy of GXP in improving the clinical symptoms and main biochemical standards of patients with coronary heart disease and the therapeutic effect in alleviating AS [30, 31]. Previous studies have shown that GXP plays an antiatherosclerotic role through multiple pathways. GXP reduces the level of NF-*κ*B, regulates the apoptosis of endothelial cells, inhibits the M1 polarization of macrophages induced by LPS, promotes M2 polarization, increases the level of serum NO, and decreases the content of plasma ET in golden hamsters with coronary AS [15, 16, 32]. Based on previous studies, we authenticate the antiatherosclerotic effect of GXP. Although some mechanisms have been studied, attention needs to be paid to the relationship between smooth muscle cells and AS in studies related to GXP. As a result, we chose VSMCs as the research object, used in combination with previous research results, and used ET-1 as the inducer.

**Figure 7:**
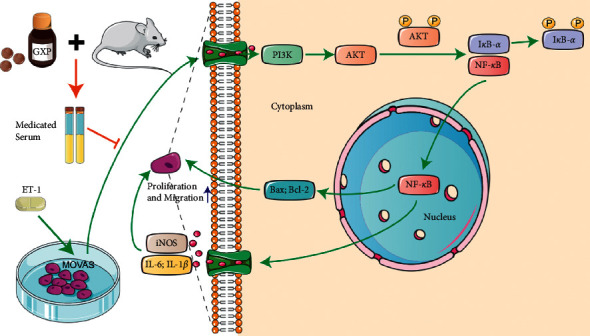
The activation of PI3K gives rise to the Akt phosphorylation, pAkt activates the transformation and decomposition of IP, which breaks down the binding with NF-*κ*B, appears as the lower expression of I*κ*B-*α*, and NF-*κ*B is more activated and transferred into the nucleus, and increases the expression of IL-6, IL-1*β*, and iNOS and upregulation of Bax/Bcl-2 ratio. Eventually, ET-1 promotes the proliferation and migration of MOVAS. Mice were given GXP tablets (2.43 g/kg·bid for 3 days) 1 h after intragastric administration on the 4th day, and under ether inhalation anesthesia, blood was taken from the abdominal aorta to obtain medicated serum. GXP may inhibit the proliferation and migration of MOVAS by inhibiting the overactivated PI3K/Akt/NF-*κ*B pathway.

In this study, we observed that the survival rate and migration rate of MOVAS in the model group treated with ET-1 were higher than those in the blank group, and PI3K/Akt/NF-*κ*B pathway was activated, which was consistent with the literature [33, 34]. GXP inhibited the proliferation and migration of MOVAS, which was remarkably induced by ET-1, and did not affect the physiological state of MOVAS. Meanwhile, GXP inhibited PI3K/Akt/NF-*κ*B pathway, which was activated by ET-1 (Figure 7). In the pathological group, GXP reduced the expression of PI3K and inhibited Akt phosphorylation; subsequently, GXP increased the expression of I*κ*B-*α*; inhibited NF-*κ*B from entering the nucleus; decreased the expression of IL-6, IL-1*β,* and iNOS; and raised the Bax/Bcl-2 ratio.

PI3K/Akt pathway is involved in cell growth, proliferation, and migration. PI3K/Akt pathway activation can lead to cell proliferation, migration, and even tumor development [35]. It is the signaling pathway of NF-*κ*B, a family of transcription factors, promotes the inflammatory response, and regulates cell proliferation and apoptosis. Moreover, the NF-*κ*B signaling pathway interacts with PI3K/Akt signaling pathway [36]. The activation of PI3K gives rise to Akt phosphorylation; further, pAkt activates the transformation and decomposition of I*κ*B-*α*, which breaks down the bond with NF-*κ*B, resulting in a lower expression of I*κ*B-*α*, activation of NF-*κ*B, and increased transfer of NF-*κ*B to the nucleus [37]. In addition, activation of PI3K/Akt signaling pathway inhibits Bax and raises the levels of proteins Bcl-2 and iNOS. Bax is the main apoptotic protein, whereas Bcl-2 is an antiapoptotic protein [38]. Inducible nitric oxide synthase (iNOS) is one of the three subtypes of NOS. High concentration of endogenous NO produced by excessive iNOS promotes cell growth and metastasis, and low concentration of iNOS inhibits cell proliferation [39].

In this study, we also observed that ET-1 can upregulate the expression of inflammation-related factors IL-6 and IL-1*β*. Therefore, we consider that ET-1 not only promotes the proliferation and migration of MOVAS but also has a certain effect on inflammation. When the pathological group with increased inflammation was treated with GXP, IL-6 and IL-1*β* were downregulated. GXP inhibited the inflammatory response of MOVAS under pathological conditions. Given that inflammation of MOVAS is also associated with *atherosclerosis*, this may also be one of the ways that GXP exerts its antiatherosclerotic effects.

GXP is a composition of five herbs, including P. sibiricum, A. sinensis, P. notoginseng (Burkill) F. H. Chen, T. kirilowii Maxim. Peel, and N. chinensis. Studies have shown that all five herbal medicines are related to the PI3K/Akt pathway and NF-*κ*B pathway. Dioscin, which was purified from *P. sibiricum*, can inhibit the PI3K/Akt pathway [40]. In contrast, a polysaccharide from *P. sibiricum* has been known for its enhancing effects on the PI3K/Akt signaling pathway [41]. In addition, studies have shown that a *Polygonatum* polysaccharide can activate the NF-*κ*B pathway [42]. Further, vascular Endothelial Growth Factor Receptor 2 (VEGFR2) activated PI3K/Akt signaling pathway can be greatly inhibited by the acetone extract of *A. sinensis* rich in phthalides [43]. Further, *Angelica sinensis* polysaccharide is a major bioactive component of *A. sinensis*. Phosphorylation levels of PI3K and Akt can be increased by *A. sinensis* polysaccharide by downregulating miR-22 in cells [44]. Besides, *A. sinensis* reduces the expression of NF-*κ*B [45]. In addition to different kinds of drug extracts, different types of *P. notoginseng* saponins also showed different effects on the PI3K/Akt pathway. A major component of *P. notoginseng (Burkill)* F. H. Chen, notoginsenoside R1, may repress miR-21's target PTEN by upregulating miR-21 and prevent the blockage of PI3K/Akt pathway [46]. In contrast, Notoginsenoside Ft1 inhibits PI3K/Akt pathway [47]. Furthermore, notoginsenoside R1 hinders NF-*κ*B pathways by modulating Toll-like receptors 4 (TLR4) [48]. In studies concerning *T. kirilowii Maxim. Peel* and *N. chinensis*, it is also mentioned that these herbs have certain effects on PI3K/Akt and NF-*κ*B pathways [49–51].

Different components in herbal medicine have different effects on PI3K/Akt and NF-*κ*B pathways, including inhibition, activation, and bidirectional regulation. GXP, which is made up of five herbs, showed inhibitory effects on PI3K/Akt/NF-*κ*B pathway, which was overactivated by ET-1 in MOVAS. This may be because of the mutual counteraction of drug components or the consistency after mutual influence. Considering the different subjects or inducers studied in various studies, it is not easy to draw a final conclusion. This may be the subtlety of the recipe. To clarify the relationship between these components, we considered that GXP should be divided into single drug components for further evaluation. In addition to the effect of GXP on the pathological state, we also observed that GXP had no intervention in the physiological group in MOVAS. Combined with previous research, we proved that smooth muscle cells have no obvious proliferation and migration in the physiological state; therefore, it is unnecessary to inhibit them [1]. This not only showed the drug safety of GXP but also showed that it targeted the pathological state in MOVAS. GXP and atorvastatin have the same effect in terms of inhibiting the proliferation and migration of MOVAS, which provides a new scheme for clinical combined prevention and treatment of *atherosclerosis* in the future.

There is another interesting discovery. In this study, we also observed that there was no significant change in TNF-*α* between groups after six independent experiments. We tested TNF-*α* in the cells again by Western blotting. There was still no band expression. Thus, for the time being, we will consider its relationship with cell characteristics of MOVAS. In follow-up experiments, we may make further observations and expect more interesting findings.

## 5. Conclusion

In this study, we confirm that GXP inhibits the proliferation and migration of MOVAS and the activation of PI3K/Akt/NF-*κ*B pathway evoked by ET-1. Meanwhile, these two factors have been known to accelerate the process of *atherosclerosis*. Therefore, we infer that GXP inhibits the proliferation and migration of VSMCs by inhibiting the activation of PI3K/Akt/NF-*κ*B pathway and finally plays an antiatherosclerotic role.

## Figures and Tables

**Table 1:** The topology parameters of key targets.

Key target	Target name	Betweenness	Closeness	Degree
INS	Insulin	0.074702	0.866197	104
IL6	Interleukin 6	0.032431	0.831081	98
AKT1	Serine-threonine protein kinase 1	0.031843	0.825503	97
VEGFA	Vascular endothelial growth factor A	0.018029	0.793548	91
TNF	Tumor necrosis factor	0.020198	0.788462	90
TP53	Tumor protein 53	0.015012	0.763975	85
EGF	Epidermal growth factor	0.021643	0.759259	84
JUN	Jun proto-oncogene	0.010376	0.754601	83

## Data Availability

Detailed data sets or any other information is available from the corresponding author upon reasonable request.

## Abbreviations

GXP: Guanxinping
AS: *Atherosclerosis*
ET-1: Endothelin-1
MOVAS: Mouse aortic vascular smooth muscle cells
VSMCs: Vascular smooth muscle cells
AKT: Protein kinase B
NF-*κ*B: Nuclear factor kappa B
I*κ*B: Inhibitor of *κ*B
BAX: Bcl-2 associated X
NO: Nitric oxide
IL: Interleukin
TNF-*α*: Tumor necrosis factor-*α*.

## References

[1] Justine B., Alessandro C., Lucie L., Pompella A., Gaucher C. (2021). Phenotypic modulation of macrophages and vascular smooth muscle cells in atherosclerosis-nitro-redox interconnections. *Antioxidants*.

[2] Chen Y., Su X., Qin Q. (2020). New insights into phenotypic switching of VSMCs induced by hyperhomocysteinemia: role of endothelin-1 signaling. *Biomedicine & Pharmacotherapy*.

[3] Sutton G., Pugh D., Dhaun N. (2019). Developments in the role of endothelin-1 in atherosclerosis: a potential therapeutic target?. *American Journal of Hypertension*.

[4] Li X., Xiao H., Lin C. (2019). Synergistic effects of liposomes encapsulating atorvastatin calcium and curcumin and targeting dysfunctional endothelial cells in reducing atherosclerosis. *International Journal of Nanomedicine*.

[5] Chen S., Dong S., Li Z. (2017). Atorvastatin calcium inhibits PDGF-*ββ*-induced proliferation and migration of VSMCs through the G0/G1 cell cycle arrest and suppression of activated PDGFR*β*-PI3K-Akt signaling cascade. *Cellular Physiology and Biochemistry*.

[6] Wang Z., Bao Z., Ding Y. (2019). N*ε*-carboxymethyl-lysine-induced PI3K/Akt signaling inhibition promotes foam cell apoptosis and atherosclerosis progression. *Biomedicine & Pharmacotherapy = Biomedecine & Pharmacotherapie*.

[7] Zhang Q., Chen L., Zhao Z. (2018). HMGA1 mediated high-glucose-induced vascular smooth muscle cell proliferation in diabetes mellitus: association between PI3K/Akt signaling and HMGA1 expression. *DNA and Cell Biology*.

[8] Linton M. F., Moslehi J. J., Babaev V. R. (2019). Akt signaling in macrophage polarization, survival, and atherosclerosis. *International Journal of Molecular Sciences*.

[9] Sivandzade F., Prasad S., Bhalerao A., Cucullo L. (2019). NRF2 and NF-*κ*B interplay in cerebrovascular and neurodegenerative disorders: molecular mechanisms and possible therapeutic approaches. *Redox Biology*.

[10] Yu X.-H., Zheng X.-L., Tang C.-K. (2015). Nuclear factor-*κ*B activation as a pathological mechanism of lipid metabolism and atherosclerosis. *Advances in Clinical Chemistry*.

[11] Tacey A., Qaradakhi T., Brennan-Speranza T., Hayes A., Zulli A., Levinger I. (2018). Potential role for osteocalcin in the development of atherosclerosis and blood vessel disease. *Nutrients*.

[12] Jia Y., Qian F., Liu Z.-H. (2011). Study on quality control standard of Guanxinping Granules. *Chinese Pharmaceutial Affairs*.

[13] Zhou Q.-M., Shao J.-D. (2007). Study on quality standards of Guanxinping tablet. *ShiZhen Journal of Traditional Chinese Medicine Research*.

[14] Zhao H. Y. (2014). *Study on Spectrum Effect Relationship and Antioxidant Mechanism of Guanxinping Preparation*.

[15] Yan S.-H., Li Q.-Y., Wang H.-D., Wan M. (2012). Effects of Guanxinping tablet containing serum on (H2) (O2)-induced apoptosis and NF-∼*κ*B expression in vascular endothelial cells. *Chinese Journal of Integrative Medicine*.

[16] Zhou G.-J., Sun Y.-T., Zhou L.-Y. (2020). Effects of Guanxinping-containing serum on RAW 264.7 macrophages polarization induced by LPS. *Journal of Nanjing University of Traditional Chinese Medicine*.

[17] Yin Y., Feng L., Wang L., Ding L. (2018). The role of curcumae rhizoma-sparganii rhizoma medicated serum in epithelial-mesenchymal transition in the triple negative breast cancer. *Biomedicine & Pharmacotherapy*.

[18] Shi B., Shi J., Qin H. (2017). Effect of medicated serum of curcumae radix extract on mRNA expression of TIMP-1, MMPs-13 and aI-collagen of HSC-T6 cell. *Saudi Pharmaceutical Journal*.

[19] Di Z.-L., Niu X.-L., Wei J., Gao D.-F., Wang N.-P. (2005). *Effect of Endothelin-1 Stimulation on Peroxisome Proliferator-Activated Receptor γ Expression in Vascular Smooth Muscle Cells*.

[20] Ding Y., Yang J., Ma Y. (2019). MYCN and PRC1 cooperatively repress docosahexaenoic acid synthesis in neuroblastoma via ELOVL2. *Journal of Experimental & Clinical Cancer Research*.

[21] Hu Y., Rao S.-S., Wang Z.-X. (2018). Exosomes from human umbilical cord blood accelerate cutaneous wound healing through miR-21-3p-mediated promotion of angiogenesis and fibroblast function. *Theranostics*.

[22] Alberts A., Klingberg A., Hoffmeister L. (2020). Binding of macrophage receptor MARCO, LDL, and LDLR to disease-associated crystalline structures. *Frontiers in Immunology*.

[23] Xu J., Zhang Z., Qian M. (2020). Cullin-7 (CUL7) is overexpressed in glioma cells and promotes tumorigenesis via NF-*κ*B activation. *Journal of Experimental & Clinical Cancer Research*.

[24] Tsai N.-P., Lin Y.-L., Tsui Y.-C., Wei L.-N. (2010). Dual action of epidermal growth factor: extracellular signal-stimulated nuclear-cytoplasmic export and coordinated translation of selected messenger RNA. *Journal of Cell Biology*.

[25] Oh S., Bournique E., Bowen D. (2021). Genotoxic stress and viral infection induce transient expression of APOBEC3A and pro-inflammatory genes through two distinct pathways. *Nature Communications*.

[26] Libby P., Buring J. E., Badimon L. (2019). Atherosclerosis. *Nature Reviews Disease Primers*.

[27] Shah A., Gray K., Figg N., Finigan A., Starks L., Bennett M. (2018). Defective Base excision repair of oxidative DNA damage in vascular smooth muscle cells promotes atherosclerosis. *Circulation*.

[28] Wang J., Uryga A. K., Reinhold J. (2015). Vascular smooth muscle cell senescence promotes atherosclerosis and features of plaque vulnerability. *Circulation*.

[29] Clement M., Raffort J., Lareyre F. (2019). Impaired autophagy in CD11b + dendritic cells expands CD4 + regulatory T cells and limits atherosclerosis in mice. *Circulation Research*.

[30] Zhang H.-B., Li Q.-Y. (2017). Effects of Guanxinping tablets on antioxidant enzymes and inflammatory factors in patients with coronary heart disease and angina pectoris. *Chinese Traditional Patent Medicine*.

[31] Han X., Li Q.-Y., Jiang M. (2014). Clinical effects and mechanisms of Guanxinping treating coronary heart disease angina pectoris. *Journal of Nanjing University of Traditional Chinese Medicine*.

[32] Wang M., Li Q.-Y., Zhu X.-X., Yan S. H., Shi L. F. (2012). The effects of Guanxinping on NO, NOS, ET, Ang II in mesocricitus auratus with atherosclerosis. *Chinese Archives of Traditional Chinese Medicine*.

[33] Planas-Rigol E., Terrades-Garcia N., Corbera-Bellalta M. (2017). Endothelin-1 promotes vascular smooth muscle cell migration across the artery wall: a mechanism contributing to vascular remodelling and intimal hyperplasia in giant-cell arteritis. *Annals of the Rheumatic Diseases*.

[34] Tian X., Zhang Q., Huang Y. (2020). Endothelin-1 downregulates sulfur dioxide/aspartate aminotransferase pathway via reactive oxygen species to promote the proliferation and migration of vascular smooth muscle cells. *Oxidative Medicine and Cellular Longevity*.

[35] Toulany M., Rodemann H. P. (2015). Phosphatidylinositol 3-kinase/Akt signaling as a key mediator of tumor cell responsiveness to radiation. *Seminars in Cancer Biology*.

[36] Balaji S., Ahmed M., Lorence E., Yan F., Nomie K., Wang M. (2018). NF-*κ*B signaling and its relevance to the treatment of mantle cell lymphoma. *Journal of Hematology & Oncology*.

[37] Coppo R. (2014). Proteasome inhibitors in progressive renal diseases. *Nephrology Dialysis Transplantation*.

[38] Wu L.-Y., Enkhjargal B., Xie Z.-Y. (2020). Recombinant OX40 attenuates neuronal apoptosis through OX40-OX40L/PI3K/AKT signaling pathway following subarachnoid hemorrhage in rats. *Experimental Neurology*.

[39] Girotti A. W., Fahey J. M. (2020). Upregulation of pro-tumor nitric oxide by anti-tumor photodynamic therapy. *Biochemical Pharmacology*.

[40] Zhang Y.-S., Ma Y.-L., Thakur K. (2018). Molecular mechanism and inhibitory targets of dioscin in HepG2 cells. *Food and Chemical Toxicology*.

[41] Zhang H., Cao Y., Chen L. (2015). A polysaccharide from polygonatum sibiricum attenuates amyloid-*β*-induced neurotoxicity in PC12 cells. *Carbohydrate Polymers*.

[42] Long T., Liu Z., Shang J. (2018). Polygonatum sibiricum polysaccharides play anti-cancer effect through TLR4-MAPK/NF-*κ*B signaling pathways. *International Journal of Biological Macromolecules*.

[43] Chen M.-C., Hsu W.-L., Chang W.-L., Chou T.-C. (2017). Antiangiogenic activity of phthalides-enriched Angelica Sinensis extract by suppressing WSB-1/pVHL/HIF-1*α*/VEGF signaling in bladder cancer. *Scientific Reports*.

[44] Pan H., Zhu L. (2018). RETRACTED: Angelica sinensis polysaccharide protects rat cardiomyocytes H9c2 from hypoxia-induced injury by down-regulation of microRNA-22. *Biomedicine & Pharmacotherapy*.

[45] Sui Y., Liu W., Tian W., Li X.-Q., Cao W. (2019). A branched arabinoglucan from Angelica sinensis ameliorates diabetic renal damage in rats. *Phytotherapy Research*.

[46] Liu Z., Wang H., Hou G., Cao H., Zhao Y., Yang B. (2019). Notoginsenoside R1 protects oxygen and glucose deprivation‐induced injury by upregulation of miR‐21 in cardiomyocytes. *Journal of Cellular Biochemistry*.

[47] Shen K., Leung S. W. S., Ji L. (2014). Notoginsenoside Ft1 activates both glucocorticoid and estrogen receptors to induce endothelium-dependent, nitric oxide-mediated relaxations in rat mesenteric arteries. *Biochemical Pharmacology*.

[48] Qian D., Shao X., Li Y., Sun X. (2019). Retracted: notoginsenoside R1 protects WI‐38 cells against lipopolysaccharide‐triggered injury via adjusting the miR‐181a/TLR4 axis. *Journal of Cellular Biochemistry*.

[49] Tuya N., Wang Y., Tong L., Gao W., Yu R., Xue L. (2017). Trichosanthin enhances the antitumor effect of gemcitabine in non-small cell lung cancer via inhibition of the PI3K/AKT pathway. *Experimental and Therapeutic Medicine*.

[50] Wang Y., Li Q., Zhou L., Ding X., Lu L. (2018). Peptide Tk-PQ induces immunosuppression in skin allogeneic transplantation via increasing Foxp3+ Treg and impeding nuclear translocation of NF-*κ*B. *Molecular Immunology*.

[51] Maiwulanjiang M., Bi C. W. C., Lee P. S. C. (2015). The volatile oil of Nardostachyos Radix et Rhizoma induces endothelial nitric oxide synthase activity in HUVEC cells. *PLoS One*.

